# Clinical, laboratory, pathological, and radiological characteristics and prognosis of patients with pulmonary salivary gland-type tumors

**DOI:** 10.1007/s00432-022-04295-5

**Published:** 2022-08-29

**Authors:** Yuan Zhang, Xiao Liu, Yumei Gu, Shu Zhang

**Affiliations:** 1 Department of Respiratory and Critical Care Medicine, Beijing Institute of Respiratory Medicine and Beijing Chao-Yang Hospital, Capital Medical University, Beijing, 100020 China; 2 Department of Radiology, Beijing Chao-Yang Hospital, Capital Medical University, Beijing, China; 3 Department of Pathology, Beijing Chao-Yang Hospital, Capital Medical University, Beijing, 100020 China

**Keywords:** Pulmonary salivary gland-type tumor, Pulmonary adenoid cystic carcinoma, Pulmonary mucoepidermoid carcinoma

## Abstract

**Purpose:**

Primary pulmonary salivary gland-type tumor (PSGT) included two main subtypes, pulmonary adenoid cystic carcinoma (PACC) and pulmonary mucoepidermoid carcinoma (PMEC). The purpose of this study was to compare the similarities and differences between these two subtypes and to identify independent risk factors for the prognosis of PSGT patients.

**Methods:**

This study screened patients with a pathological diagnosis of PSGT in Beijing Chaoyang Hospital between 2010 and 2021. The clinical, pathological, radiological, laboratory test, and other characteristics were collected, and *t*, nonparametric and chi-squared tests were used to compare the differences in clinical characteristics of the two subtypes. COX univariate and multivariate analyses were used to explore prognostic-related risk factors.

**Results:**

A total of 62 patients with PSGT were included in our center over a 12-year period. There were 26 PMEC patients and 36 PACC patients. There were differences in the clinical, pathological, and radiological features of the two tumor subtypes. Univariate analysis showed that weight loss, chemotherapy, white blood cells, lymphocytes, red blood cells, total protein, and total bilirubin might be related to the prognosis in PSGT patients. Multivariate results showed that lymphocytes (*p* = 0.031), red blood cells (*p* = 0.047), total protein (*p* = 0.032), and total bilirubin (*p* = 0.010) were independent prognostic risk factors. Chemotherapy (HR 4.452; 95% CI 1.723–11.503; *p* = 0.002) might be associated with progression-free survival (PFS).

**Conclusion:**

The two subtypes of PSGT had significantly different clinical, laboratory, pathological, and radiological features. However, there was no significant difference in the prognosis of patients with PMEC and PACC subtypes. Cox univariate and multivariate analyses showed that levels of lymphocytes, erythrocytes, total protein and total bilirubin in the peripheral blood of PSGT patients might be related to patient overall survival. Chemotherapy might also be associated with PFS.

## Introduction

Primary pulmonary salivary gland-type tumor (PSGT) is one kind of rare tumors originating from the submucosal glands of the trachea and bronchus (Sauter et al. 2021; Goode et al. 1998). Its incidence only accounts for only 0.09% to 0.20% of the total incidence of lung cancer (Molina et al. 2007). It includes pulmonary adenoid cystic carcinoma (PACC), pulmonary mucoepidermoid carcinoma (PMEC), epithelial myoepithelial carcinoma, clear cell carcinoma, carcinoma ex pleomorphic adenoma, and so on (Moran 1995). Among them, mucoepidermoid carcinoma and adenoid cystic carcinoma are the two most common salivary gland tumors, accounting for approximately 90% (Sauter et al. 2021; Falk et al. 2016). Among them, the incidence of PMEC is slightly higher than that of PACC (Kumar et al. 2018).

PMEC and PACC, as two main subtypes of PSGT, have similar biological behavior due to the same type of tumor stroma. However, there are still differences in its internal clinical characteristics, pathological characteristics, and therapeutic effects, and its specific similarities and differences remain to be further clarified. Previous studies have shown that the prognosis of PACC might be significantly better than that of PMEC (Kumar et al. 2018). Therefore, the first aim of this study was to elucidate the clinical, pathological, and radiological differences between PACC and PMEC.

Due to its low incidence, previous studies on PSGT of the lung were mostly case reports or small sample studies. At present, the analysis of factors related to its prognosis is still very limited. The prognosis of patients with PSGT is markedly heterogeneous. Some studies suggest that the duration of complaint, tumor size, and treatment may be independent prognostic factors for primary tracheobronchial adenoid cystic carcinoma (Wang et al. 2019) Surgical treatment of PMEC patients with only N stage identified can affect patient outcomes (Wang et al. 2020). Based on the degree of cellular differentiation, atypia, and mitosis, PMEC is classified into low-grade and high-grade (Wang et al. 2015). In contrast to low-grade PMEC, high-grade PMEC is usually composed of solid squamous cells. Moreover, high-grade PMEC is also more prone to recurrence and metastasis (Roden et al. 2022; Zhu et al. 2013; Chen et al. 2014).

At the time of diagnosis, the peripheral blood before treatment can reflect the inflammatory state or immunosuppressive state of the patient to a certain extent, which may affect the prognosis of the patient. However, whether peripheral blood indicators affect the prognosis of PSGT patients is still unknown. The second purpose of this study was to investigate the prognostic role of various laboratory markers in peripheral blood.

## Materials and methods

### Study population

We first collected data from patients diagnosed with primary salivary gland-type tumors of the lung in Beijing Chaoyang Hospital between June 1, 2010 and December 31, 2021. Inclusion criteria were pathologically confirmed primary salivary gland-type tumors of the lung and the age was over 18 years old. Exclusion criteria were missing information or comorbidity with other primary malignancies. Patients were screened according to the inclusion and exclusion criteria. This study was approved by the Ethics Committee of Beijing Chaoyang Hospital (No. 2009–4).

### Clinical and laboratory data

The clinical data and laboratory test data were collected from the electronic medical record database for all enrolled patients. Clinical data mainly included age, sex, Eastern Cooperative Oncology Group Performance Status (ECOG PS), body mass index (BMI), weight loss, smoking history, diagnostic methods, and tumor location of the patients at the time of diagnosis.

The second part that needed to be focused on was the laboratory variables of peripheral blood before treatment, including whole blood cell analysis, biochemical indicators, and some tumor markers at the time of diagnosis. Specifically, the indicators in the whole blood cell analysis included leukocyte count, neutrophil count, lymphocyte count, eosinophil count, basophil count, monocyte count, erythrocyte count, hemoglobin levels, and platelet count. Specific blood biochemical indicators included total protein, prealbumin, albumin, globulin, lactate dehydrogenase, alkaline phosphatase, aspartate transaminase, alanine transaminase, triglycerides, cholesterol, total bilirubin, direct bilirubin, creatinine, potassium, sodium, chlorine, and glucose. Tumor markers mainly focused on carcinoembryonic antigen and carbohydrate antigen 125.

### Histology and immunohistochemical staining

Hematoxylin & eosin (H&E) and immunohistochemical staining were performed on 4-μm-thick formalin-fixed paraffin-embedded tissue sections. The Ventana HE 600 automated staining system (Ventana Medical Systems, Inc., Tucson, AZ) was used for H&E staining. Original H&E slides were rereviewed by two pathologists to confirm the original pathological diagnosis in each case. Immunohistochemical staining were performed with anti-cytokeratin 7 (CK7) antibody (MX-053, mouse monoclonal), anti-P63 antibody (MX013, mouse monoclonal), and anti-c-kit (CD117) antibody (YR145, rabbit monoclonal) using a Ventana Bench Mark ULTRA Autostainer (Ventana Medical Systems, Inc., Tucson, AZ). All the antibodies mentioned above were from FUZHOU MAIXIN BIOTECH, China. Immunohistochemical staining was evaluated by two pathologists blinded to clinical information.

### Imaging technique and image analysis

The scanning equipment was a third-generation, dual-source computer tomography (CT) (SOMATOM Definition Force; Siemens Healthcare, Forchheim, Germany). The contrast agent used was Iopamiro ® (iohexol containing 370 mg/mL iodine, Bracco Diagnostics). All CT images were reconstructed into a slice thickness of 5.0 mm. The scans were performed in the supine position during end-inspiration. At the beginning of the scan, patients were asked to hold their breath while the image of the chest was obtained, with a scanning range from the thoracic entrance to the level of the diaphragm. The GE picture archiving and communication system was used for film reading. These images were first assessed by a mid-career radiologist and then reviewed and revised by a senior radiologist.

### Treatment and follow-up strategies

The main treatment options collected in this study included surgery, radiotherapy and chemotherapy. All patients undergoing surgery or treatment were recommended to have a routine check-up at least once a year. For patients who had not undergone complete surgery or treatment, examinations were recommended at least every 6 months. The follow-up information of all enrolled patients was collected from the electronic medical record system. Finally, all patients were followed up by telephone on April 1, 2022. The patient's treatment, recurrence and prognosis information in other hospitals would be recorded in detail. The primary endpoint of this study was the prognosis of the patient. Overall survival was defined as the time from diagnosis of PSGT to death or the end of follow-up. Progression-free survival (PFS) was defined as the time interval between the time of diagnosis and the first confirmation of local or distant recurrence or death or the end of follow-up.

### Statistical analysis

Categorical data were summarized as frequencies and percentages, and nonnormally distributed continuous variables were represented by the median and interquartile range (IQR). Other continuous variables were shown as the mean ± standard deviation (SD). The chi-squared test was used to compare categorical variables between two groups, and the *t* test or one-way ANOVA test was used to compare continuous variables between two groups according to normality and homogeneity of variance. Survival curves were estimated by the Kaplan–Meier method and compared using the log-rank test. Potential factors affecting survival were estimated using a multivariate Cox proportional hazards regression model. Additional analyses were performed by SPSS software (version 25.0; IBM, Armonk, NY, USA). Professional epidemiologists reviewed the statistical methods of this article.

## Results

### Patient characteristics

In this study, 65 patients diagnosed with PSGT were screened through the inclusion criteria, but 3 patients met the exclusion criterion of incomplete information, so 62 patients were finally included in this study. Among them were 34 men, with a median age of 55 and a median BMI of 23.410. At diagnosis, 93.5% (*n* = 58) of patients had an ECOG PS score of 0, and 85.5% (*n* = 53) of patients had no significant weight loss. 41.9% (*n* = 26) of the patients were former smokers. The diagnosis was confirmed by surgery in 58.1% (*n* = 36) of the patients. A total of 41.9% (*n* = 26) of patients were diagnosed by bronchoscopy. 27.4% (*n* = 17) of patients received chemotherapy, and 72.6% (*n* = 45) did not. Eight patients received radiation therapy. Among them, there were six PACC patients and two PEMC patients. Of the six patients diagnosed with PACC, five received postoperative adjuvant prophylactic radiotherapy and one received palliative radiotherapy. Two PMEC patients received palliative radiotherapy (Table 1).

**Table 1:** Baseline demographic and clinical characteristics of the study population

Characteristic	Whole patients (*n* = 62)	PMEC (*n* = 26)	PACC (*n* = 36)	*P* value
Age (years)	55.0 (43.0, 60.0)	59.5 (46.8, 65.0)	51.0 (38.5, 57.75)	0.021
Gender				0.368
Male	34 (54.8)	16 (61.5)	18 (50.0)	
Female	28 (45.2)	10 (38.5)	18 ( 50.0)	
ECOG PS				0.079
0	58 (93.5)	26 (100.0)	32 (88.9)	
1–3	4 (6.4)	0 (0)	4 (11.1)	
BMI (kg/m^2^)	23.410 (21.975, 26.368)	23.550 (22.035, 24.725)	23.145 (21.003, 27.183)	0.792
Weight loss				0.572
No	53 (85.5)	23 (88.5)	30 (88.3)	
Yes	9 (14.5)	3 (11.5)	6 (16.7)	
Smoke history				0.106
Never	36 (58.1)	12 (46.2)	24 (66.7)	
Current and former	26 (41.9)	14 (53.8)	12 (33.3)	
Surgery				0.002
No	26 (41.9)	5 (19.2)	21 (58.3)	
Yes	36 (58.1)	21 (80.8)	15 (41.7)	
Tumor location				< 0.001
Trachea/mainstem bronchi/ lobar /segmental bronchi	41 (66.1)	10 (38.5)	31 (86.1)	
Other location	21 (33.9)	16 (61.5)	5 (13.9)	
Lymphadenopathy				0.023
No	41 (66.1)	13 (50.0)	28 (77.8)	
Yes	21 (33.9)	13 (50.0)	8 (22.2)	
Airway obstruction				0.023
No	41 (66.1)	13 (50.0)	28 (77.8)	
Yes	21 (33.9)	13 (50.0)	8 (22.2)	
Crescent sign				0.013
No	29 (46.8)	17 (65.4)	12 (33.3)	
Yes	33 (53.2)	9 (34.6)	24 (66.7)	
Radiotherapy				0.298
No	54 (87.1)	24(92.3)	30 (83.3)	
Yes	8 (12.9)	2(7.7)	6 (16.7)	
Chemotherapy				0.026
No	45 (72.6)	15 (57.7)	30 (83.3)	
Yes	17(27.4)	11 (42.3)	6 (16.7)	
Leukocyte (10^9^/L)	7.337 ± 2.621	7.451 ± 2.312	6.440 (5.475,8.198)	0.400
Neutrophil (10^9^/L)	3.900 (2.988, 5.470)	4.667 ± 1.912	3.485 (2.845,5.313)	0.220
Lymphocyte (10^9^/L)	1.983 ± 0.728	2.034 ± 0.847	1.946 ± 0.639	0.640
Eosinophil (10^9^/L)	0.125 (0.060, 0.205)	0.140 (0.088,0.280)	0.142 ± 0.131	0.096
Basophil (10^9^/L)	0.020 (0.010, 0.040)	0.020 (0.010,0.040)	0.020 (0.013, 0.040)	0.982
Monocyte (10^9^/L)	0.480 ± 0.327	0.487 ± 0.207	0.400 (0.313, 0.540)	0.288
Erythrocyte (10^9^/L)	4.449 ± 0.513	4.348 ± 0.578	4.380 (4.268, 4.833)	0.146
Hemoglobin (g/L)	130.890 ± 20.636	129.730 ± 21.813	134.500 (127.000, 142.750)	0.412
Platelet (10^9^/L)	242.840 ± 60.094	252.460 ± 85.457	235.890 ± 54.641	0.390
Total protein (g/L)	67.394 ± 7.135	68.115 ± 7.205	66.000(63.825, 72.475)	0.318
Prealbumin (g/L)	0.227 ± 0.075	0.225 ± 0.094	0.228 ± 0.058	0.884
Albumin (g/L)	38.876 ± 4.730	38.038 ± 5.423	39.481 ± 4.135	0.261
Globulin (g/L)	28.519 ± 5.411	30.077 ± 6.260	27.394 ± 4.466	0.069
Lactate dehydrogenase (U/L)	168.600 ± 46.030	156.500 (137.750, 186.255)	162.000 (141.000,180.000)	0.559
Alkaline phosphatase (U/L)	80.320 ± 26.119	89.620 ± 29.488	73.610 ± 21.387	0.016
Aspartate transaminase (U/L)	19.000 (16.000, 23.250)	19.500 (16.000, 23.250)	18.000 (16.000, 23.750)	0.652
Alanine transaminase (U/L)	17.000 (15.000, 25.000)	17.00 (14.750, 25.250)	17.000 (15.250, 24.750)	0.836
Triglycerides (mmol/L)	0.975 (0.748, 1.633)	0.995 (0.713, 1.953)	0.950 (0.760, 1.238)	0.613
Cholesterol (mmol/L)	4.530 (3.893, 4.870)	4.286 ± 0.852	4.498 ± 0.744	0.301
Total bilirubin (umol/L)	10.800(8.050, 14.225)	9.690 ± 3.594	11.425 ± 4.808	0.016
Direct bilirubin (umol/L)	3.145 ± 1.416	2.660 ± 1.177	3.496 ± 1.484	0.021
Creatinine (umol/L)	65.666 ± 16.510	69.600 ± 15.156	62.825 ± 17.064	0.111
Potassium (mmol/L)	3.865 ± 0.558	3.900(3.600, 4.125)	3.861 ± 0.543	0.524
Sodium (mmol/L)	140.069 ± 3.324	140.011 ± 3.975	140.111 ± 2.823	0.955
Chlorine (mmol/L)	104.206 ± 3.436	104.108 ± 3.350	104.700 (103.025,105.975)	0.373
Glucose (mmol/L)	4.875 (4.28, 5.43)	4.985 (4.245, 6.175)	4.805 (44.373,5.378)	0.603
Carcinoembryonic antigen (ng/ml)	2.230 (1.065, 4.868)	3.730 (1.303, 8.850)	1.875 (0.955,2.473)	0.019
Carbohydrate antigen 125 (U/ml)	14.770 (9.245, 19.243)	15.930 (12.600, 43.978)	11.175 (7.680,17.680)	0.004

*PACC* pulmonary adenoid cystic carcinoma, *PMEC* pulmonary mucoepidermoid carcinoma, *ECOG PS* Eastern Cooperative Oncology Group performance status, *BMI* body mass index

### Comparison of PMEC and PACC patient demographics and clinical characteristics

A total of 26 PMEC cases (41.9%) and 36 PACC cases (58.1%) were included in this study. Sex, ECOG PS, BMI, weight loss, smoking history, leukocytes, neutrophils, lymphocytes, eosinophils, basophils, monocytes, erythrocytes, hemoglobin, platelets, total protein, prealbumin, albumin, globulin, lactate dehydrogenase, aspartate transaminase, alanine transaminase, triglycerides, cholesterol, creatinine, potassium, sodium, chlorine, and glucose were not significantly different between the two tumor types. However, age (*p* = 0.021), tumor location (*p* < 0.001), chemotherapy (*p* = 0.026), alkaline phosphatase (*p* = 0.016), total bilirubin (*p* = 0.016), direct bilirubin (*p* = 0.021), carcinoembryonic antigen (*p* = 0.019), and carbohydrate antigen 125 (*p* = 0.004) showed significant differences between the two subtypes. There was a highly significant difference between the two groups for surgical treatment (*p* = 0.002), which may greatly impact their survival (Table 1).

### Comparison of PMEC and PACC patient pathological features

**Fig. 1:**
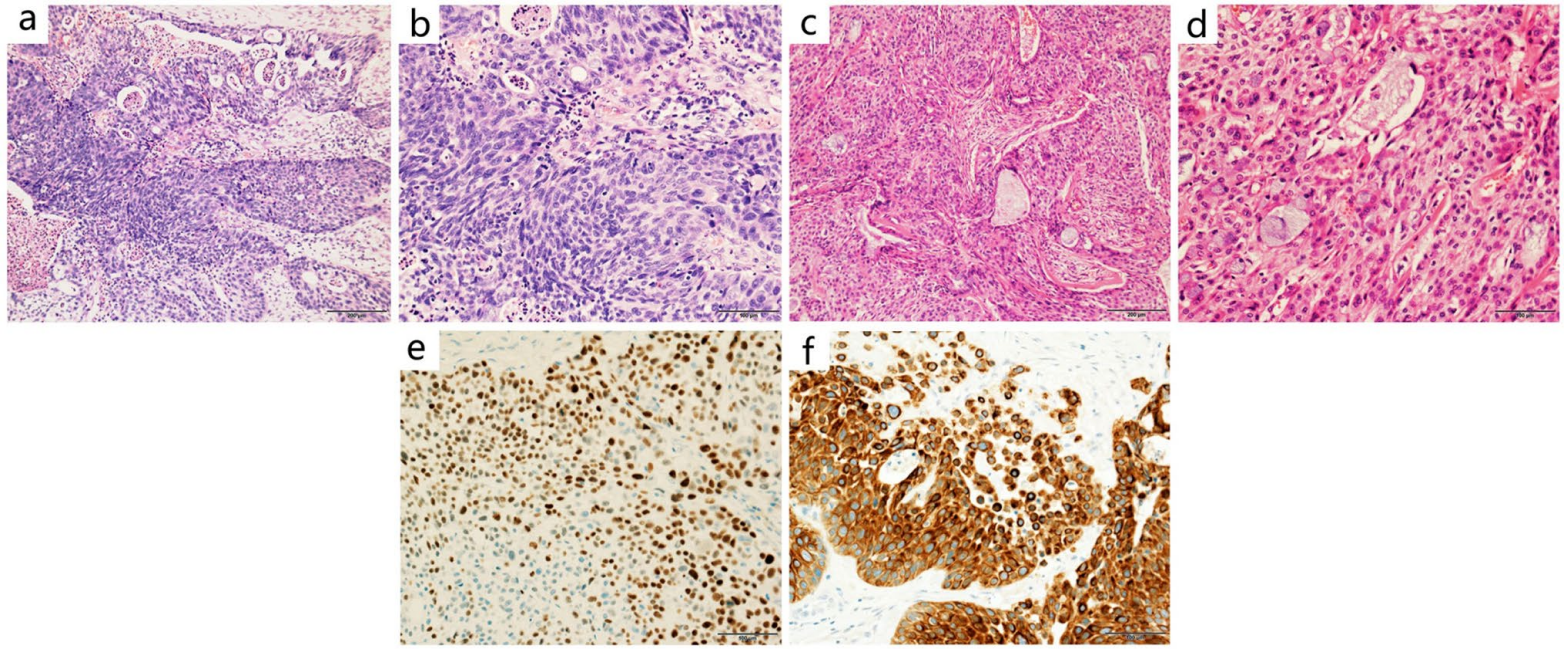
Microscopic images and immunostaining of primary pulmonary mucoepidermoid carcinoma. **a** (Low power at 10 ×) and **b** (high power 20 ×) microscopic images showed the features for a case of high-grade PMEC, including atypical epidermal and intermediate cells, with fewer mucinous cells. With readily visible mitoses and marked cellular atypia and necrosis. **c** (Low power at 10 ×) and **d** (high power 20 ×) microscopic images showed the features for a case of low-grade PMEC, including glands, tubules, cysts, and solid areas, the majority of which were glandular tubes with mucus-secreting cells. **e**, **f** Show immunohistochemical staining for P63 and CK7. p63 and CK7 were positive in PMEC cases. p63 was positive in both intermediate and epidermoid cells

In our PMEC group, gross pathological examination revealed that the lesion diameters ranged from 0.4 to 8 cm. Lesions were grayish white and the boundaries were unclear. Microscopic observation indicated that the cancer tissue was composed of mucous cells, epidermoid cells, and intermediate cells. A total of 26 cases were categorized into two groups based on their cell proportions and atypia as follows: (1) High grade: 11 cases (11/26), consisted mainly of atypical epidermal and intermediate cells, with fewer mucinous cells. With readily visible mitoses and marked cellular atypia and necrosis (Fig. 1a, b). (2) Low grade: 15 cases (15/26), consisted of a collection of glands, tubules, cysts, and solid areas, the majority of which were glandular tubes with mucus-secreting cells. Intermediate and epidermal-like cells were scattered inside a solid nest. Intercellular bridges and keratosis could be found in epidermal-like cells. The abovementioned cells were well differentiated, with no or rare mitotic figures and necrosis (Fig. 1c–d).

Immunohistochemical staining was negative for TTF-1 and Napsin A, and positive for P63. (Fig.1e–f). MAML2 gene rearrangement was detected by in situ hybridization.

**Fig. 2:**
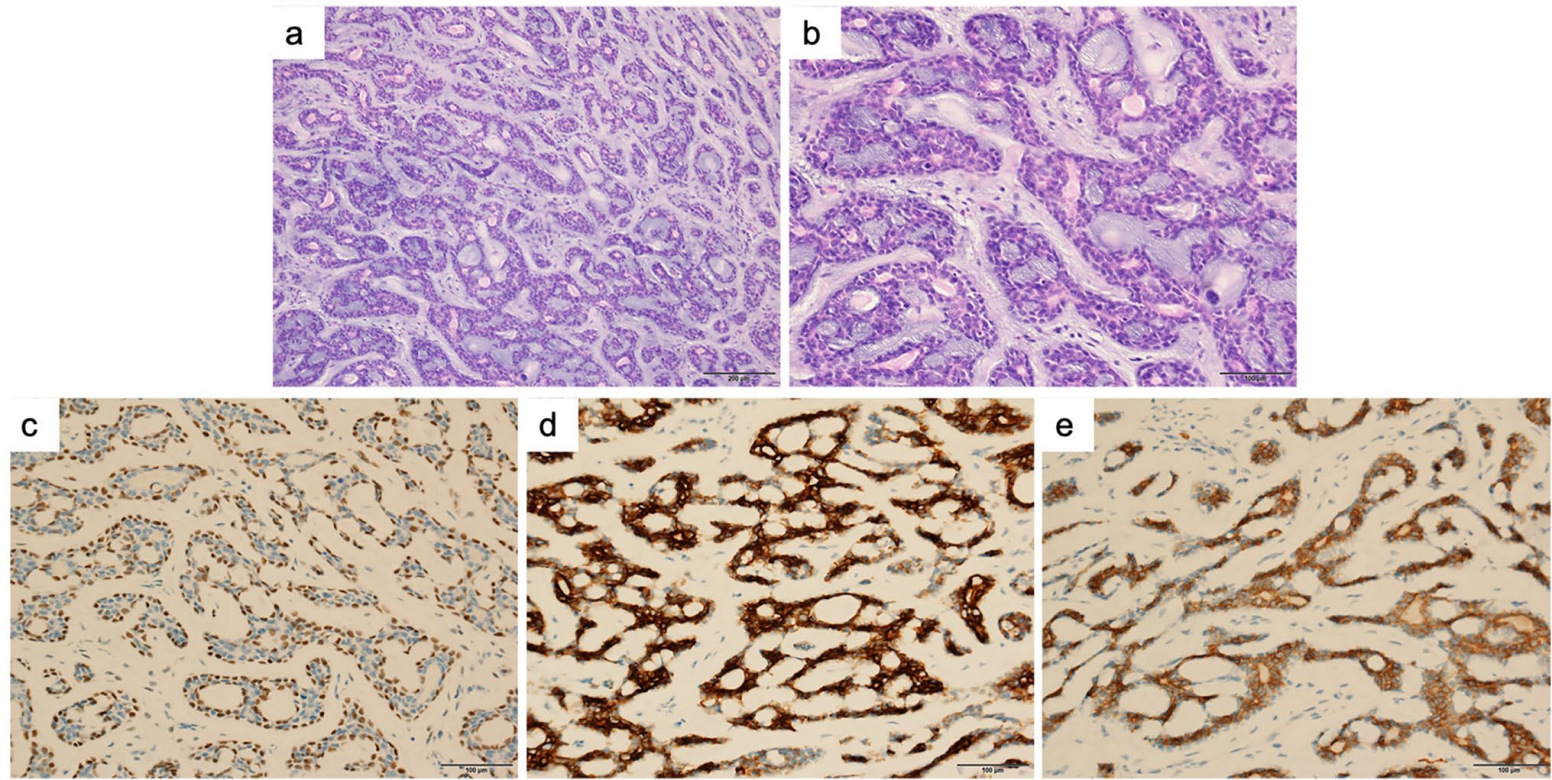
Microscopic images and immunostaining of pulmonary adenoid cystic carcinoma. **a** (Low power at 10 ×) and **b** (high power 20 ×) showed the features of a case of pulmonary adenoid cystic carcinoma. Tumors were composed of epithelial and myoepithelial cells. Epithelial cells were in the inner layer of glandular, tubular and cribriform structures, and myoepithelial cells were in the outer layer. **c**, **d**, and** f** showed the immunohistochemical staining for P63, CK7, and CD117. p63 was expressed in myoepithelial cells, whereas internal luminal cells were always negative. CK7 was expressed in internal luminal cells. CD117 was expressed in both internal luminal cells and myoepithelial cells

In the PACC group, the lesion diameters ranged from 0.8 to 6.8 cm. Lesions were also grayish white and the boundaries were unclear. The tumor cells of the PACC were small with hyperchromatic nuclei. Arranged in sieve, glandular, cord-like, and solid nests. A dilated pseudocyst could be seen inside. Mucus or eosinophilic basement membrane-like material could be seen in the cyst. Tumors were composed of epithelial and myoepithelial cells. It was most evident in glandular, tubular, and cribriform structures. Epithelial cells were in the inner layer of glandular, tubular and cribriform structures, and myoepithelial cells were in the outer layer (Fig. 2a–b). The epithelial cells showed positive staining for CK, EMA and CD117. The myoepithelial cells showed positive staining for SMA and P63 (Fig. 2c–e).

### Comparison of PMEC and PACC patient radiological features

Tumors were found in the trachea or mainstem bronchi or lobar or segmental bronchi (41/62, 66.1%) and other location (21/62, 33.9%). PACCs were more common in the trachea or bronchi than PMECs (86.1% vs. 38.5%). Of the 10 patients with PMEC in the trachea or bronchi, intraluminal (8/10, 80%), and extraluminal (2/10, 20%) extensions were observed on CT. Among the 31 patients with PACC in the trachea or bronchi, intraluminal extensions with (8/31, 25.8%) or without (19/31, 61.3%) extraluminal extensions and extraluminal extensions only (1/31, 3.2%) were identified. Involvement of infiltrative wall-thickening lesions with (24/31, 77.4%) or without (5/31, 16.1%) focal nodules. Thirty patients showed a crescent-shaped gap around the tumor (“air crescent sign”). Tumors were round to oval (8/62, 12.9%), lobulated (15/62, 24.2%), or had circumferential thickening (39/62, 62.9%). PMECs were mainly round to oval (7/26, 26.9%), but most PACCs were lobulated or had circumferential thickening (35/36, 97.2%). Associated CT findings suggestive of airway obstruction disease were found in 18 patients (PMEC = 10 and PACC = 8) and included distal bronchial regions with obstructive pneumonia (9/18, 50.0%) and subsegmental atelectasis (6/18, 33.3%). Mediastinal or hilar lymph node enlargement was observed in both PMEC (10/26, 38.5%) and PACC (8/36, 22.2%) (Table 1).

**Fig. 3:**
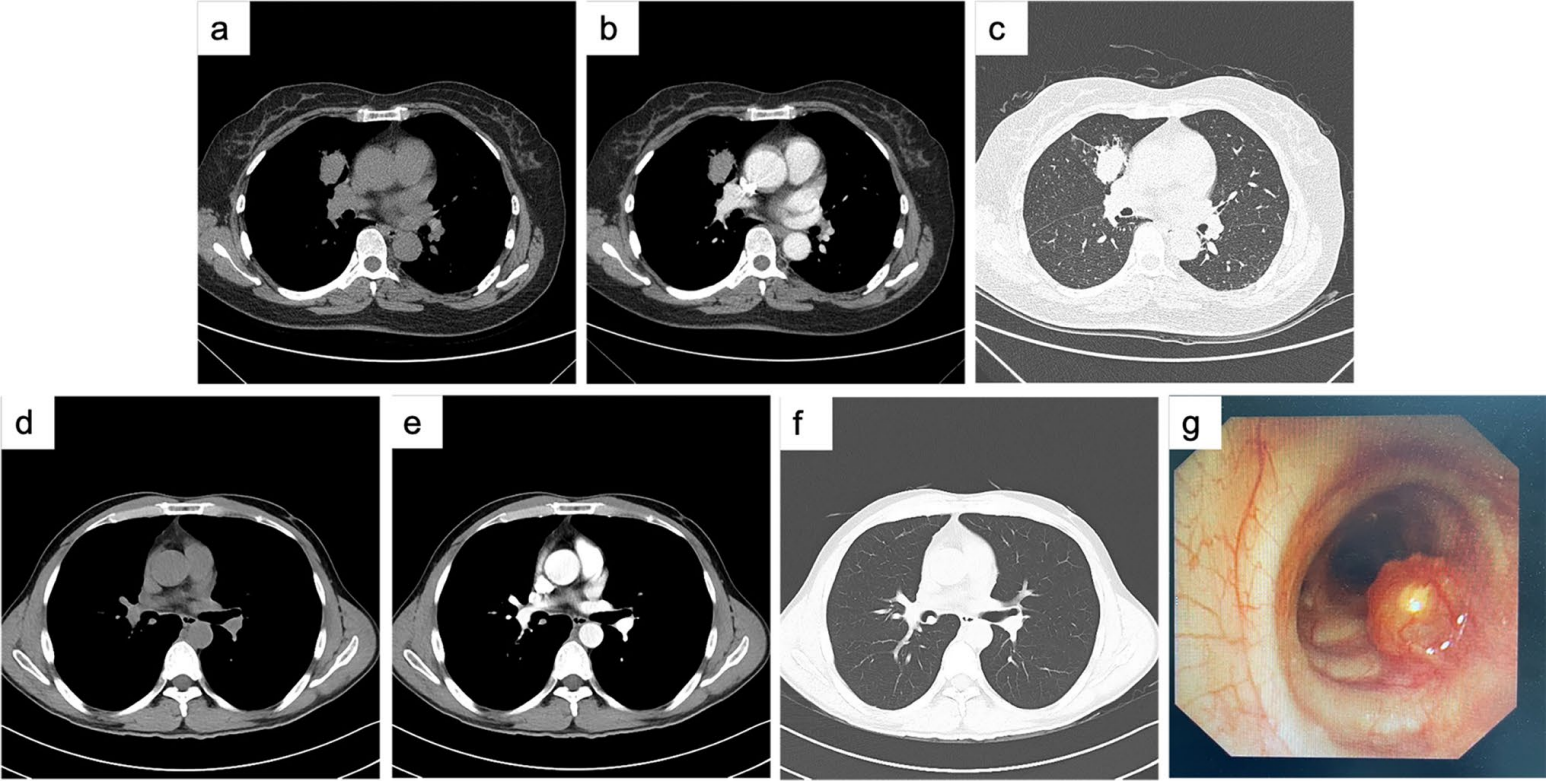
CT and bronchoscopy images of pulmonary mucoepidermoid carcinoma of peripheral type (**a**–**c**) and central type (**d**–**g**). **a**, **d** Plain transverse image of mediastinal window; **b**, **e** Enhanced transverse image of mediastinal window; **c**, **f** Transverse image of the lung window; **g** Bronchoscopy images

The peripheral PMEC was a soft tissue mass with a maximum cross-section of 1.9*3.2 cm in the medial subsegment of the anterior segment of the right upper lobe. Local bronchial continuity is interrupted. Patchy ground-glass opacities in the distal lung parenchyma. Consolidation with beaded bronchial dilatation. The consolidation part showed mild to moderate enhancement after enhancement (Fig. 3a–c). Chest CT showed that the central PMEC manifested as a nodule with a diameter of approximately 0.7 cm in the right middle bronchus. Its edges are smooth, and the center is less dense. Punctate calcification was seen at the posterior edge, and mild enhancement was seen (Fig. 3d–f). Bronchoscopy revealed a spherical neoplasm in the right middle bronchus. The surface was slightly congested, and yellow‒white nodules could be seen inside. The nodules were hard in texture. No abnormality was found in the bronchi of other lobe segments (Fig. 3g).

**Fig. 4:**
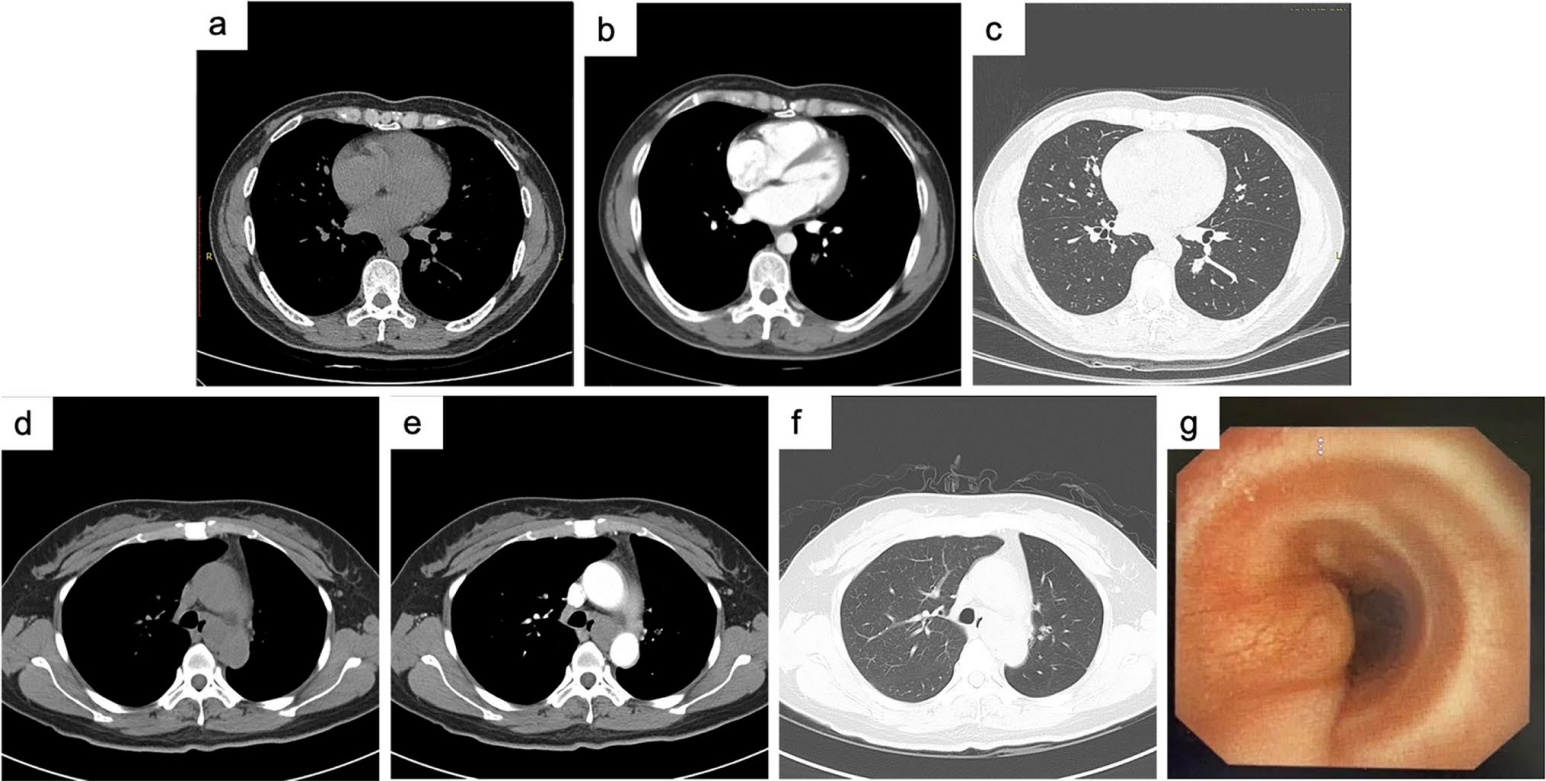
CT and bronchoscopy images of pulmonary adenoid cystic carcinoma of peripheral type **a**–**c** and central type **d**–**g**. **a**, **d** Plain transverse image of mediastinal window; **b**, **e** Enhanced transverse image of mediastinal window; **c**, **f** Transverse image of the lung window; **g** Bronchoscopy images

The peripheral-type PACC included in this study showed a nodule with a size of approximately 0.9 × 1.6 cm in cross-section in the dorsal segment of the left lower lobe on chest CT. Distributed along bronchovascular bundles, with slightly shallow lobulated margins. The distribution of bronchioles could be seen in the lesion, and the corresponding bronchial cavity was narrowed and occluded. Mild enhancement was seen with enhancement (Fig. 4a–c). The central PACC we included showed a local mass with a cross-section of approximately 3.2*2.3 cm, which blocked the left main bronchus and protruded into the left wall of the trachea. Enhanced scans showed enhancement (Fig. 4d–f). Bronchoscopy revealed neoplastic obstruction at the left main opening with a broad base. The surface was smooth and tough. Bleeding less during biopsy (Fig. 4g).

### Cutoff values for laboratory variables at diagnosis for survival analysis

Normality calculations were performed for all hematological parameters, such as leukocytes, lymphocytes, monocytes, erythrocytes, hemoglobin, platelets, total protein, prealbumin, albumin, globulin, lactate dehydrogenase, alkaline phosphatase, direct bilirubin, creatinine, potassium, sodium, and chlorine, which are expressed as the mean ± SD. Nonnormally distributed continuous variables, such as neutrophils, eosinophils, basophils, aspartate transaminase, alanine transaminase, triglycerides, cholesterol, total bilirubin, glucose, carcinoembryonic antigen, and carbohydrate antigen 125, were expressed as the median and IQR. Available hematological indices of complete blood cytology, biochemistry, and tumor markers were listed in Table 1. According to the ROC curve, we calculated cutoff values for all laboratory parameters, such as the cutoff value of leukocytes, which was 7.630. All patients were divided into two groups according to the cutoff value. There were 39 patients (62.9%) in the low group (leukocyte ≤ 7.630). There were 23 patients (37.1%) in the high group (leukocyte > 7.630). Other laboratory indicators were calculated in the same way (Table 2).

**Table 2:** Univariable and multivariable Cox regression analyses between baseline characteristics, laboratory variables, and overall survival or progression-free survival of patients with primary salivary gland-type tumors of the lung

Characteristic	No. (%)	Overall survival				Progression-free survival			
		Univariate analysis		Multivariable analysis		Univariate analysis		Multivariable analysis	
		χ2	*P* value	HR (95% CI)	*P* value	χ2	*P* value	HR (95% CI)	*P* value
Age (years)									
≤ 55	32 (51.6)	1		1		1		1	
> 55	30 (48.4)	1.536	0.466	1.157 (0.198–6.763)	0.871	1.626	0.289	1.337 (0.501–3.566)	0.562
Gender									
Male	34 (54.8)	1		1		1		1	
Female	28 (45.2)	0.560	0.346	9.053 (0.799–102.608)	0.075	0.776	0.580	1.344 (0.480–3.761)	0.574
ECOG PS									
0	58 (93.5)	1				1			
1–3	4 (6.5)	1.065	0.915			1.605	0.529		
BMI (kg/m^2^)									
≤ 23.99	38 (61.3)	1				1			
> 23.99	24(38.7)	2.273	0.163			0.924	0.862		
Weight loss									
No	53 (85.5)	1		1		1			
Yes	9 (14.5)	6.45	0.003	2.720 (0.535–13.833)	0.228	2.166	0.139		
Smoke history									
Never	36 (58.1)	1				1			
Current and former	26 (41.9)	2.024	0.230			1.074	0.874		
Surgery									
No	26 (41.9)	1				1			
Yes	36 (58.1)	0.453	0.179			0.702	0.429		
Tumor location									
Trachea/mainstem bronchi/ lobar /segmental bronchi	38 (61.3)	1				1			
Other location	24 (38.7)	1.854	0.287			1.219	0..666		
Lymphadenopathy									
No	41 (66.1)	1				1			
Yes	21 (33.9)	2.717	0.090			1.654	0.365		
Airway obstruction									
No	41 (66.1)	1				1			
Yes	21 (33.9)	3.032	0.074			1.305	0.559		
Crescent sign									
No	19 (46.8)	1				1			
Yes	33 (53.2)	0.370	0.105			0.785	0.589		
Histology									
PMEC	26 (41.9)	1				1			
PACC	36 (58.1)	1.065	0.915			1.434	0.444		
Radiotherapy									
No	54 (87.1)	1				1			
Yes	8 (12.9)	0.447	0.442			0.928	0.906		
Chemotherapy									
No	45 (72.6)	1		1		1		1	
Yes	17(27.4)	4.365	0.012	4.153 (0.715–24.137)	0.113	4.292	0.001	4.452 (1.723–11.503)	0.002
Leukocyte (10^9^/L)									
≤ 7.630	39 (62.9)	1		1		1			
> 7.630	23 (37.1)	4.731	0.020	7.137 (0.973–52.330)	0.053	1.504	0.363		
Neutrophil (10^9^/L)									
≤ 6.130	49 (79.0)	1				1			
> 6.130	13 (21.0)	3.075	0.056			1.270	0.644		
Lymphocyte (10^9^/L)									
≤ 2.560	51(82.3)	1		1		1			
> 2.560	11 (17.7)	3.300	0.042	10.579 (1.237–90.478)	0.031	1.068	0.906		
Eosinophil (10^9^/L)									
≤ 0.240	49 (79.0)	1				1			
> 0.240	13 (21.0)	0.496	0.507			0.838	0.780		
Basophil (10^9^/L)									
≤ 0.025	36 (58.1)	1				1			
> 0.025	26 (41.9)	0.489	0.284			0.454	0.127		
Monocyte (10^9^/L)									
≤ 0.475	35 (56.5)	1				1			
> 0.475	27 (43.5)	1.421	0.551			0.822	0.665		
Erythrocyte (10^9^/L)									
≤ 4.285	24(38.7)	1		1		1			
> 4.285	38(61.3)	0.257	0.027	0.150 (0.023–0.977)	0.047	0.844	0.712		
Hemoglobin (g/L)									
≤ 142.5	46 (74.2)	1				1			
> 142.5	16 (25.8)	1.431	0.561			1.583	0.329		
Platelet (10^9^/L)									
≤ 241.500	33 (53.2)	1				1			
> 241.500	29 (46.8)	0.313	0.086			0.707	0.450		
Total protein (g/L)									
≤ 67.250	34 (54.8)	1		1		1			
> 67.250	28 (45.2)	5.130	0.018	22.199 (1.315–374.714)	0.032	1.348	0.509		
Prealbumin (g/L)									
≤ 0.225	33 (53.2)	1				1			
> 0.225	29 (46.8)	0.609	0.418			1.018	0.969		
Albumin (g/L)									
≤ 37.950	27 (43.5)	1				1			
> 37.950	35 (56.5)	0.980	0.973			0.917	0.851		
Globulin (g/L)									
≤ 27.650	30 (48.4)	1				1			
> 27.650	32(51.6)	4.330	0.060			0.958	0.925		
Lactate dehydrogenase (U/L)									
≤ 149.500	23 (37.1)	1				1			
> 149.500	39 (62.9)	0.764	0.649			1.432	0.456		
Alkaline phosphatase (U/L)									
≤ 72.500	24 (38.7)	1				1			
> 72.500	38(61.3)	2.931	2.931			1.849	0.235		
Aspartate transaminase (U/L)									
≤ 10.500	2 (3.2)	1				1			
> 10.500	60 (96.8)	0.728	0.765			24.201	0.448		
Alanine transaminase (U/L)									
≤ 24.500	46 (74.2)	1				1			
> 24.500	16 (25.8)	2.081	0.214			2.065	0.115		
Triglycerides (mmol/L)									
≤ 1.130	37 (59.7)	1				1			
> 1.130	25 (40.3)	0.348	0.174			0.751	0.558		
Cholesterol (mmol/L)									
≤ 5.385	56 (90.3)	1				1			
> 5.385	6 (9.7)	2.560	0.160			1.370	0.615		
Total bilirubin (umol/L)									
≤ 15.250	50(80.6)	1		1		1			
> 15.250	12 (19.4)	3.773	0.031	11.209 (1.780–70.607)	0.010	2.014	0.159		
Direct bilirubin (umol/L)									
≤ 2.535	25 (40.3)	1				1			
> 2.535	37 (59.7)	0.581	0.360			0.908	0.831		
Creatinine (umol/L)									
≤ 88.700	55 (88.7)	1				1			
> 88.700	7 (11.3)	3.048	0.070			1.457	0.503		
Potassium (mmol/L)									
≤ 4.150	44 (71.0)	1				1			
> 4.150	18 (29.0)	2.319	0.153			1.697	0.262		
Sodium (mmol/L)									
≤ 137.250	10 (16.1)	1				1			
> 137.250	52 (83.9)	0.461	0.213			0.632	0.377		
Chlorine (mmol/L)									
≤ 105.250	39 (62.9)	1				1			
> 105.250	23 (37.1)	0.544	0.361			0.658	0.391		
Glucose (mmol/L)									
≤ 4.910	32 (51.6)	1				1			
> 4.910	30 (48.4)	1.977	0.266			1.162	0.739		
Carcinoembryonic antigen (ng/ml)									
≤ 3.155	45 (72.6)	1				1			
> 3.155	17 (27.4)	2.388	0.132			1.212	0.682		
Carbohydrate antigen 125 (U/ml)									
≤ 15.570	38 (61.3)	1				1			
> 15.570	24 (38.7)	2.893	0.084			1.583	0.308		

*PACC* pulmonary adenoid cystic carcinoma, *PMEC* pulmonary mucoepidermoid carcinoma, *HR* hazard ratio, *CI* confidence interval, *ECOG PS* Eastern Cooperative Oncology Group performance status *BMI* body mass index

### Survival analysis

The median follow-up time was 50.5 months. Due to the low mortality rate, the median survival time could not be calculated at this time. Thirteen patients died during follow-up. Among them, 6 patients had PMEC. Seven patients had PACC. The mortality rates were 23.1% and 19.4%, respectively. Among these patients. The shortest surviving patient died 1 month after diagnosis. The patient who survived the longest died 136 months after diagnosis.

The half-year survival rate of all patients was 96.8%, the 1-year survival rate was 91.5%, the 2-year survival rate was 88.7%, the 5-year survival rate was 85.5%, and the 10-year survival rate was 80.6%. The half-year survival rate of PMEC patients was 96.2%, the 1-year survival rate was 88.5%, the 2-year survival rate was 84.6%, the 5-year survival rate was 84.6%, and the 10-year survival rate was 80.8%. The half-year survival rate of PACC patients was 97.2%, the 1-year survival rate was 94.4%, the 2-year survival rate was 91.7%, the 5-year survival rate was 86.1%, and the 10-year survival rate was 80.6%. There was no significant difference in the prognosis of the two groups of patients (*p* = 0.915).

**Fig. 5:**
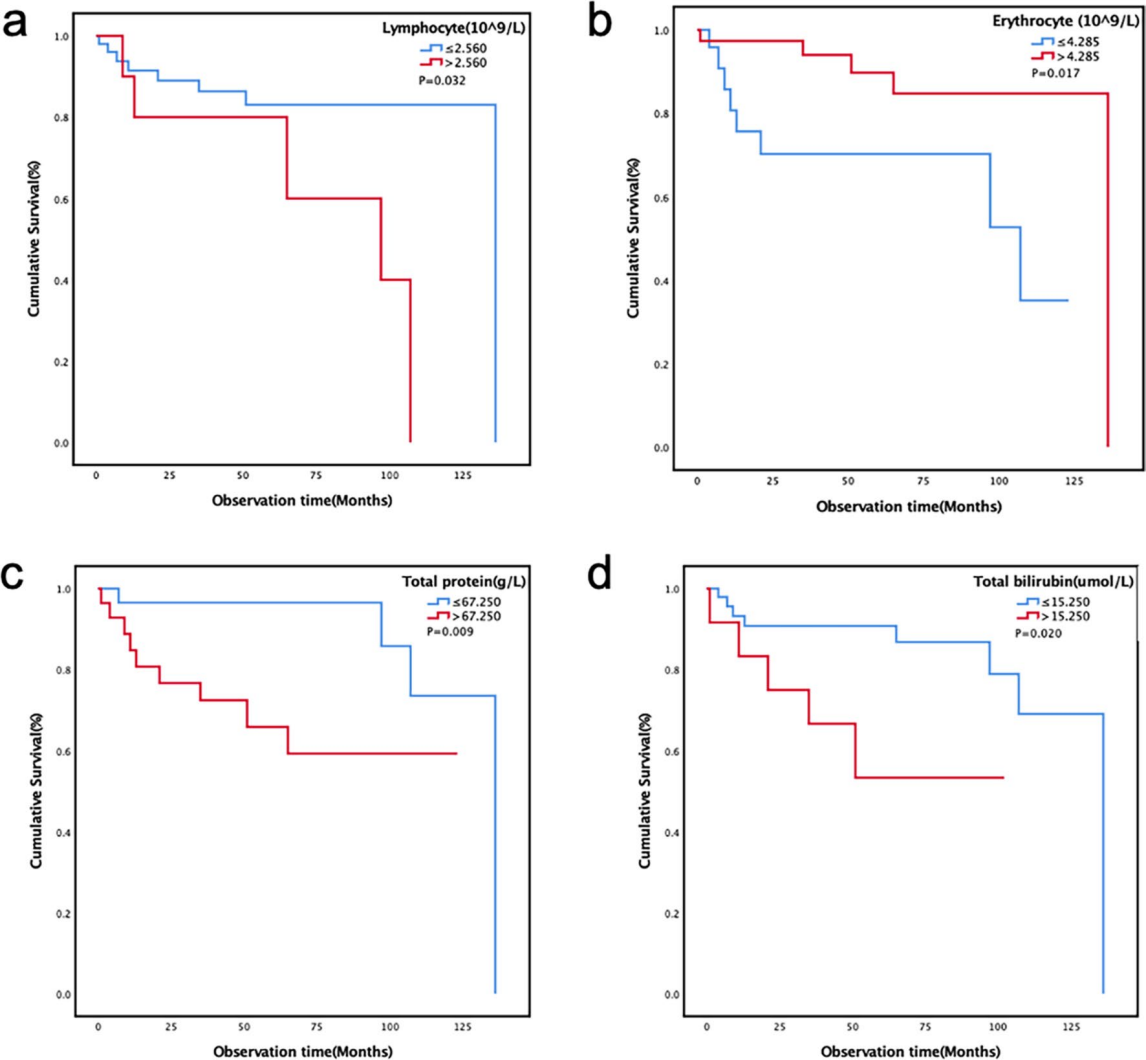
Kaplan–Meier plots of overall survival in the lung primary salivary gland-type tumor patients according to (a) lymphocyte; (**b**) erythrocyte; (**c**) total protein; (**d**) total bilirubin groups

The Cox univariate analysis showed that weight loss (*p* = 0.003), chemotherapy (*p* = 0.012), leukocytes (*p* = 0.020), lymphocytes (*p* = 0.042), erythrocytes (*p* = 0.027), total protein (*p* = 0.0180), and total bilirubin (*p* = 0.031) might be associated with prognosis. Other indicators were not associated with prognosis (Table 2). The multivariate analysis included age, sex, and indicators significantly associated with prognosis in univariate analysis. The results showed that lymphocytes [hazard ratio (HR), 10.579; 95% confidence interval (CI) 1.237–90.478; *p* = 0.031], erythrocytes (HR 0.150; 95% CI 0.023–0.977; *p* = 0.047), total protein (HR 22.199; 95% CI 1.315–374.714; *p* = 0.032), and total bilirubin (HR 11.209; 95% CI 1.780–70.607; *p* = 0.010) were independent risk factors for prognosis (Table 2). Patients in the high-erythrocyte group had a better prognosis than those in the low-erythrocyte group (Fig. 5b). Compared with the low-lymphocyte group, the low total protein group and the low total bilirubin group, the high-lymphocyte group (Fig. 5a), the high total protein group (Fig. 5c) and the high total bilirubin group (Fig. 5d) had a worse prognosis.

During the follow-up period, 14 patients (22.6%) had recurrence or metastasis, 4 patients were PMEC and 10 patients were PACC. The recurrence and metastasis rates were 15.4% and 27.8%, respectively. Among these patients, the patients with the shortest recurrence or metastasis time were found 1 month after diagnosis. The patients with the longest recurrence or metastasis time were found 64 months after diagnosis.

For all patients, the half-year recurrence rate was 6.5%, the 1-year recurrence rate was 9.7%, the 2-year recurrence rate was 16.1%, the 5-year recurrence rate was 21.0%, and the 10-year recurrence rate was 22.6%. The half-year recurrence rate of PMEC patients was 3.8%, the 1-year recurrence rate was 7.7%, and the 2-year recurrence rate was 15.4%. The half-year recurrence rate of PACC patients was 8.3%, the 1-year recurrence rate was 11.1%, the 2-year recurrence rate was 16.7%, the 5-year recurrence rate was 25.0%, and the 10-year recurrence rate was 27.8%.

**Fig. 6:**
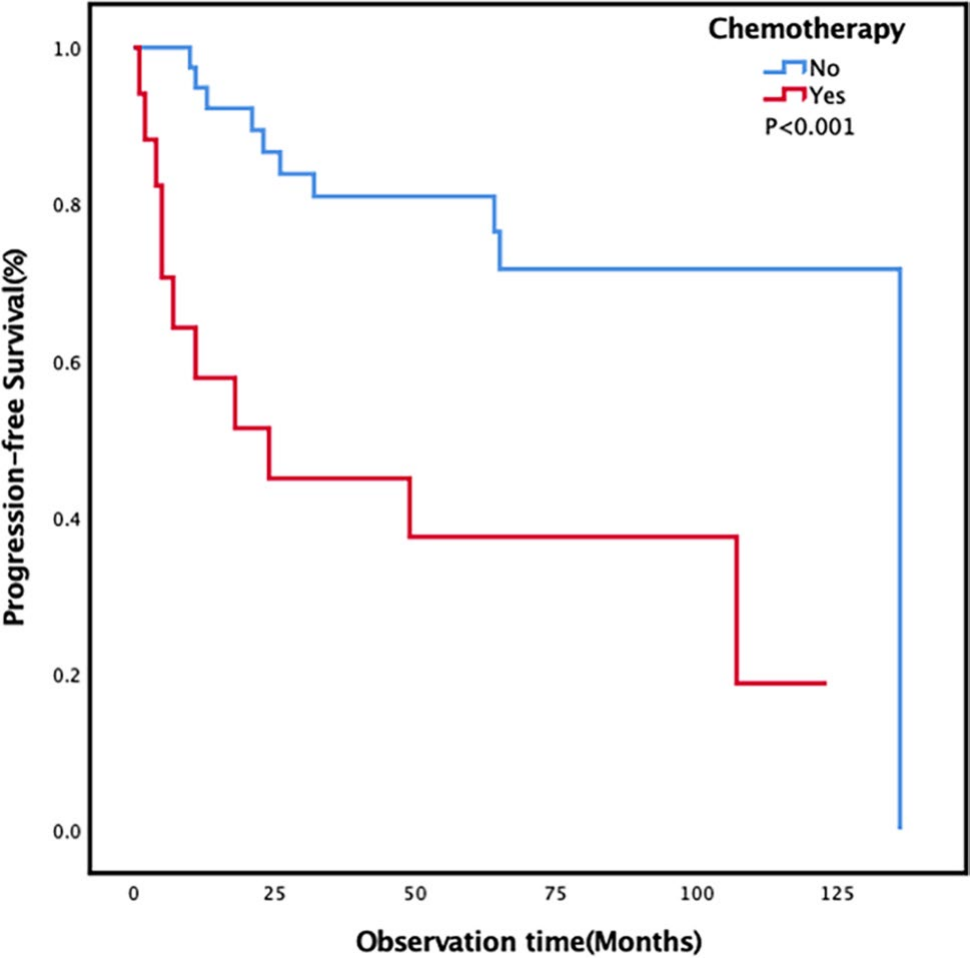
Progression-free survival curves in the lung primary salivary gland-type tumor patients stratified by chemotherapy

The Cox univariate and multivariate analyses showed that chemotherapy (HR 4.452; 95% CI 1.723–11.503; *p* = 0.002) might be associated with PFS (Fig. 6). Other indicators were not related to PFS (Table 2).

## Discussion

This retrospective study mainly focused on patients with PSGT. We collected as many clinical, pathological, radiological and laboratory indicators of patients as possible. The differences in clinical characteristics and prognosis of PMEC and PACC patients were comprehensively analyzed. The prognostic risk factors for PSGT patients were further identified by survival analysis.

### Similarities and differences between the two tumor subtypes

The median age of PSGT in our center was 55 years. Compared with PMEC patients, PACC patients were younger, which was inconsistent with previous studies (Molina et al. 2007; Wang et al. 2020). There were more PACC patients than PMEC patients in this study, similar to previous studies (Wang et al. 2020; Li et al. 2018; Kim et al. 2020). Other previous studies had shown that PMEC was slightly more common than PACC (Kumar et al. 2018; Wang et al. 2021). There was still controversy as to which tumor subtype was more common. We speculated that the proportion of the two tumors might be related to different areas. PACC was more likely to grow in the trachea and main bronchus, so only some patients received reconstruction of the trachea, and many others received subsequent chemotherapy consistent with previous research (Molina et al. 2007). At the same time, more patients with TMEC in segmental bronchi were more likely to undergo pneumonectomy.

Pathological examination suggested that low-grade PMEC consisted of epidermal cells, mucous cells, and intermediate cells. Cystic adenoid and solid nest structure. High-grade PMEC were mainly composed of atypical epidermal and intermediate cells, with prominent mitoses and marked cellular atypia. PACC was usually composed of two cellular components, glandular and myoepithelial. Arranged in sieve, glandular, cord-like, and solid nests. The observations of this study were consistent with previous studies (Roden et al. 2022; Puzyrenko et al. 2021; Cheng et al. 2017).

Imaging analysis of all PSGT patients were performed in our study. The following characteristics were summarized, which were similar to other previous studies (Li et al. 2018; Cheng et al. 2017; Ban et al. 2021; Han et al. 2019). PSGT tumors were mainly divided into the intraluminal polyp, intraluminal and extraluminal mass, full-thickness infiltration, and peripheral mass types. Most PMECs were observed in segmental or lobar bronchi and appearred as sharply well-defined, either ovoid or lobulated, intraluminal nodules that adapted to the branching features of the airways. The shape was mostly oval or lobulated. The "air crescent sign" could be seen in the bronchi. Peripheral PMEC was rare, and CT findings were not characteristic. Malignant signs such as lobulated burrs might appear. Necrotic cavities were common. PACCs were more common in the lower trachea or mainstem bronchi and present with circumferential thickening or intraluminal and extraluminal extension. Long luminal stenosis caused by infiltrative growth along the vessel wall was more common than in PMEC. Most of the images showed uniform low density, intratumoral calcification was rare, and the enhancement was not obvious after enhancement. Obvious enhancement was more commonly observed in PMEC than in PACC. Lymph node metastasis was rare in both tumors.

Focusing on laboratory test indicators, PACC had lower alkaline phosphatase, carcinoembryonic antigen, and carbohydrate antigen 125 levels. However, PACC had higher total bilirubin and direct bilirubin. These features might also suggest the type of histological subtype before diagnosis.

### Prognostic risk factors for salivary gland tumors

At present, the exploration of prognostic risk factors in patients with PSGT were mainly limited to clinical, radiological (Han et al. 2019), pathological, and treatment factors (Lichtenberger et al. 2018), but relatively little attention had been given to peripheral blood cells and biochemical indicators. In this study, Cox multivariate analysis showed that lymphocytes, erythrocytes, total protein and total bilirubin were significantly correlated with the prognosis of patients with PSGT. Lymphocytes were crucial components of the adaptive immune system (Rabinowich et al. 1987).Previous studies had shown that patients with malignant PSGT had lower mean lymphocyte counts and percentages than patients with benign SGT (Damar et al. 2016). In addition, other studies had shown that the neutrophil-to-lymphocyte ratio might serve as a useful prognosticator for a high risk of multiple recurrences in patients with adenoid cystic carcinoma (Brkic et al. 2019) and primary parotid mucoepidermoid carcinoma (Gao et al. 2020). There were few studies on the correlation between erythrocytes, total protein and total bilirubin and prognosis. Their prognostic role in patients with PSGT still needed to be further verified.

In this study, chemotherapy mainly included two parts. In addition to conventional postoperative adjuvant chemotherapy, it also included therapeutic chemotherapy after subsequent recurrence. If the patient needed postoperative adjuvant chemotherapy, it suggested that the patient might have a high malignant potential or risk factors. If a patient developed recurrence and metastases during follow-up, therapeutic chemotherapy was reasonable at that time. In conclusion, receiving chemotherapy indicated that the PFS of patients was poor.

This study’s results suggested that PMEC patients had higher mortality than PACC patients. However, the recurrence rate of PACC was higher than that of PMEC. Previous studies had shown that PSGT had weak invasiveness and a slow growth rate (Wang et al. 2021; Kang et al. 2011). Therefore, the incidence of recurrence and metastasis in patients was low, so it was generally considered to be a low-grade malignant tumor (Kumar et al. 2018; Resio et al. 2018). The comparison of survival between the two tumor types was still controversial. Some studies suggested that the 5-year survival rate of PACC patients was better than that of PMEC patients, the prognosis was similar at 7–8 years, and the prognosis was worse after that (Kumar et al. 2018). However, another group of researchers showed that the prognosis of PMEC patients was similar to that of PACC patients within 5 years after diagnosis, and after 5 years, the prognosis of PMEC patients was better than that of PACC patients (Resio et al. 2018). Therefore, large-scale prospective studies were required to explore the prognosis for the two subtypes.

Secondly, there was no significant correlation between the two tumor types and prognosis, which was consistent with previous studies (Kumar et al. 2018; Kim et al. 2020; Qin et al. 2018). The prognosis of patients was affected by a variety of confounding factors, such as the malignant potential of the tumor type itself and the corresponding treatment. Although both types were inert tumors, they still had the possibility of recurrence and metastasis. The treatment procedures for salivary gland tumors were multidisciplinary and multimodal. The primary treatment option was surgery. Whether operation or not was the key factor influencing the prognosis and recurrence. In this study, PMEC patients were more likely to receive surgical treatment than PACC patients. Therefore, the recurrence rate of PACC was higher than that of PMEC. However, surgery was also a double-edged sword. As a kind of traumatic surgery, surgery would damage the immune system of patients to a certain extent, affected the lung function of patients, and bring potential surgical complications. Patients who could not tolerate surgery could choose bronchoscopy to relieve symptoms of dyspnea (Kim et al. 2020). If surgery or bronchoscopic intervention were not suitable, chemotherapy and/or radiation therapy were performed according to the patient's condition. For some special patients, tyrosine kinase inhibitor therapy (Macarenco et al. 2022; Xie et al. 2020) and immunotherapy (Gelsomino et al. 2020) were also gradually developing. Therefore, there was no significant correlation between tumor type and prognosis, which might be affected by other factors.

### Limitations

This retrospective study still had many limitations. First, lung salivary gland tumors were very rare, and this study was performed in a single center, so the number of patients enrolled was very small. Bias was very difficult to avoid. Second, the patients in this study were included from a large time span, and the treatment plans might be different, which might increase the heterogeneity of the data to a certain extent. Third, because some patients had not undergone surgery, large pathological specimens could not be obtained. Therefore, characteristics such as pathological grading could not be accurately determined. Since some tumors grew in the main airways, accurate tumor staging cold not be determined.

## Conclusion

In conclusion, PSGT was a very rare tumor with a good prognosis. Some clinical, laboratory, pathological and imaging characteristics of PACC and PMEC were different, but the prognosis was not significantly different. The results of this study showed that laboratory parameters detected in peripheral blood, such as levels of lymphocytes, erythrocytes, total protein and total bilirubin, might be significant prognostic factors. Chemotherapy might be associated with PFS. However, the above results still needed verification through more large-sample prospective studies.

## Data Availability

The datasets generated during and/or analyzed during the current study are available from the corresponding author on reasonable request.
